# Cost-effectiveness of nivolumab plus gemcitabine-cisplatin as first-line treatment for advanced urothelial carcinoma in China and the United States

**DOI:** 10.3389/fimmu.2024.1426024

**Published:** 2024-09-13

**Authors:** Guiyuan Xiang, Yueyue Huang, Lanlan Gan, Linning Wang, Yunqi Ding, Yuanlin Wu, Haiyan Xing, Yao Liu

**Affiliations:** ^1^ Department of Pharmacy, Daping Hospital, Army Medical University, Chongqing, China; ^2^ Department of Operation Management, Women and Children’s Hospital of Chongqing Medical University, Chongqing, China; ^3^ Department of Operation Management, Chongqing Health Center for Women and Children, Chongqing, China; ^4^ School of International Pharmaceutical Business, China Pharmaceutical University, Nanjing, China; ^5^ Department of Oncology, Daping Hospital, Army Medical University, Chongqing, China

**Keywords:** nivolumab, urothelial carcinoma, cost-effectiveness, gemcitabine, cisplatin, chemotherapy, partitioned survival model

## Abstract

**Objective:**

Nivolumab, recently proven in a phase 3 clinical trial (CheckMate 901) to enhance survival when combined with gemcitabine-cisplatin for advanced urothelial carcinoma. This study aimed to assess its cost-effectiveness against gemcitabine-cisplatin alone, from US and Chinese payers’ perspectives.

**Methods:**

A partitioned survival model was established to assess the life-years, quality-adjusted life-years (QALYs), lifetime costs, and incremental cost-effectiveness ratios (ICERs) of nivolumab plus gemcitabine-cisplatin versus gemcitabine-cisplatin alone as first-line treatment for advanced urothelial carcinoma. Univariate, two-way, and probabilistic sensitivity analyses were conducted to assess the model’s robustness. Additionally, subgroup analyses were performed.

**Results:**

Nivolumab plus gemcitabine-cisplatin and gemcitabine-cisplatin achieved survival benefits of 4.238 life-years and 2.979 life-years for patients with advanced urothelial carcinoma, respectively. Compared with gemcitabine-cisplatin, nivolumab plus gemcitabine-cisplatin resulted in ICERs of $116,856/QALY in the US and $51,997/QALY in China. The probabilities of achieving cost-effectiveness at the current willingness-to-pay thresholds were 77.5% in the US and 16.5% in China. Cost-effectiveness could be reached if the price of nivolumab were reduced to $920.87/100mg in China. Subgroup analyses indicated that the combination had the highest probability of cost-effectiveness in patients under 65 or with an Eastern Cooperative Oncology Group (ECOG) performance-status score of 0 in the US and China.

**Conclusion:**

Nivolumab plus gemcitabine-cisplatin first-line treatment for advanced urothelial carcinoma results in longer life expectancy than gemcitabine-cisplatin, but is not cost-effective in China at current price. However, cost-effectiveness is likely to be achieved in most patient subgroups in the US.

## 1 Introduction

Cancer represents a significant public health challenge worldwide. Among these, bladder cancer stands as the world’s tenth most prevalent cancer and ranks sixth among males (1, 2). Urothelial carcinoma is the predominant histological type of bladder cancer and the most frequent malignancy within the urinary tract (3). Chemotherapy, primarily cisplatin-based, has served as the cornerstone of first-line treatment for unresectable or metastatic urothelial carcinoma over the past four decades (4, 5). In recent years, studies have shown that immune checkpoint inhibitors can markedly enhance survival outcomes for patients with urothelial carcinoma (6, 7), thus offering innovative therapeutic alternatives for managing this advanced-stage malignancy.

Platinum-based drugs can induce immunomodulatory effects and thus thereby enhancing the efficacy of immune checkpoint blockade, which provides theoretical support for the combination therapy of programmed death-1/ligand-1 (PD-1/L1) inhibitors with platinum-based chemotherapy in treating advanced urothelial carcinoma (5, 8). Nivolumab is a humanized immunoglobulin G4 monoclonal antibody that targets PD-1 (9). The phase 3 randomized trial, CheckMate 901, assessed the therapeutic efficacy and safety of nivolumab plus gemcitabine-cisplatin versus gemcitabine-cisplatin alone in patients with untreated, unresectable, or metastatic urothelial carcinoma (9). The results demonstrated that adding nivolumab to gemcitabine-cisplatin significantly improved overall survival (OS, 21.7 months vs 18.9 months; hazard ratio [HR], 0.78; 95% confidence interval [CI], 0.63-0.96) and progression-free survival (PFS, 7.9 months vs 7.6 months; HR, 0.72; 95% CI, 0.59-0.88) in patients with urothelial carcinoma. However, it also increased the incidence of grade 3 or higher adverse events when combined with chemotherapy (61.8% versus 51.7%) (9).

While the addition of nivolumab to chemotherapy improved treatment outcomes for patients with advanced urothelial carcinoma, it concurrently elevated the incidence of adverse events. Moreover, the significantly higher cost of nivolumab compared to gemcitabine-cisplatin substantially increases the treatment cost per cycle for patients. This introduces considerable economic uncertainty for those suffering from advanced urothelial carcinoma. This study aimed to evaluate the cost-effectiveness of nivolumab plus gemcitabine-cisplatin as a first-line treatment for advanced urothelial carcinoma, from the perspectives of payers in the US and the healthcare system in China.

## 2 Methods

### 2.1 Model overview

**Figure 1:**
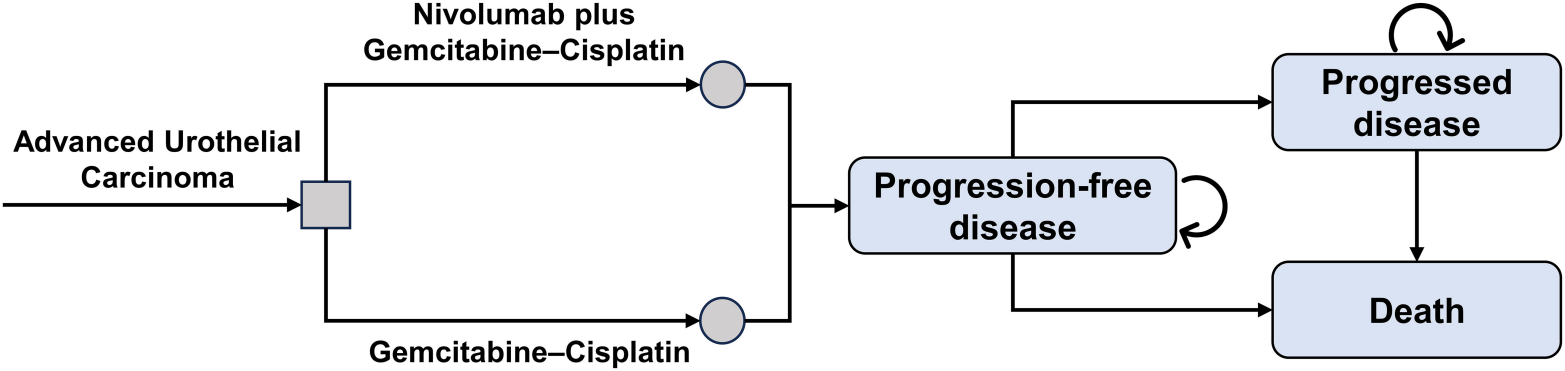
Simplified partitioned survival model.

Partitioned survival models are among the most commonly utilized modeling approaches in pharmacoeconomic evaluations, especially for the economic evaluation of oncology therapies (10). We developed a partitioned survival model using Microsoft Excel 2019 (Redmond, Washington, US) that includes three health states: PFS, progressive disease (PD), and death. Patients entered the model in the PFS state and transitioned to the PD state or death after treatment with nivolumab and/or gemcitabine-cisplatin, with these state transitions being irreversible (**Figure 1**). The model cycle length was set at three weeks, simulating a lifetime horizon. Health outcomes for the nivolumab group and the chemotherapy group were measured in quality-adjusted life-years (QALYs) and life-years, with the incremental cost-effectiveness ratio (ICER) as the primary measure for assessing the cost-effectiveness of the two treatment options. Based on the recommendations of the Institute for Clinical and Economic Review and published literature, this study set the willingness-to-pay threshold for the US at $150,000 per QALY (11–13). For China, the willingness-to-pay threshold was determined according to the WHO-CHOICE guidelines and the China Guidelines for Pharmacoeconomic Evaluations, set at three times the per capita gross domestic product, or $38,043 per QALY (14–16). An annual discount rate of 3% for the US perspective and 5% for China was applied to both costs and health outcomes (17, 18). This research was conducted following the Consolidated Health Economic Evaluation Reporting Standards (CHEERS) reporting guideline (19) (**Supplementary Table 1**).

### 2.2 Patient cohort

This study simulated a hypothetical patient cohort with baseline characteristics and treatment regimens consistent with the CheckMate 901 trial (9). A total of 608 adult patients with unresectable or metastatic urothelial carcinoma (median age 65 years) were randomly assigned in a 1:1 ratio (304:304) to either the nivolumab group or the chemotherapy group. The chemotherapy group received gemcitabine (1000 mg/m^2^, days 1 and 8 of each cycle) plus cisplatin (70 mg/m^2^, day 1 of each cycle) every three weeks for up to six cycles. The nivolumab group received nivolumab (360 mg, day 1 of each cycle) plus gemcitabine and cisplatin for up to six cycles every three weeks, followed by nivolumab treatment at a dose of 480 mg every four weeks until disease progression, unacceptable toxic effects, withdrawal of consent, death, or up to a maximum of 24 months. According to the CheckMate 901 trial, the median duration of treatment in the nivolumab and chemotherapy groups was 7.4 and 3.7 months, respectively (9). After disease progression, patients received second-line treatment, followed by best supportive care until death, with the proportion of second-line treatment drugs shown in **Supplementary Table 2**. Given that pembrolizumab is not approved by the NMPA for urothelial carcinoma and avelumab is not available in China, this study assumes that patients in China would participate in clinical trials as an alternative to using pembrolizumab and avelumab in subsequent treatments (20).

### 2.3 Clinical efficacy data inputs

We initially extracted time and survival rate data from the OS and PFS survival curves of the CheckMate 901 trial using the WebPlotDigitizer program (version 4.6, https://automeris.io/WebPlotDigitizer/). Following the method of Guyot et al. (21), we generated pseudo-individual patient data using R software (version 4.3.0, http://www.r-project.org) to reconstruct the survival curves. We then fitted exponential, Weibull, gamma, Gompertz, log-logistic, lognormal, and generalized gamma parametric models. Model fit was assessed based on the Akaike information criterion (AIC) and Bayesian information criterion (BIC), with smaller values indicating a better fit (AIC and BIC results are shown in **Supplementary Table 3**). The best-fitting parametric model for the OS survival curves of both the nivolumab group and the chemotherapy group, as well as the PFS curve of the chemotherapy group, was the log-logistic distribution. The best-fitting parametric model for the PFS curve of the nivolumab group was the generalized gamma distribution (**Table 1**). Survival curves were extrapolated to the point where 99% of patients had died, resulting in a time horizon of 30 years. In this study, to minimize the error in survival data of the simulated cohort in the partitioned survival model, survival rate data within 60.2 months for both treatment groups were derived from the survival curves of the CheckMate 901 trial. The fitted survival curves using the parametric models are shown in **Supplementary Figure 1**.

**Table 1:** Basic clinical and health parameters: baseline values, ranges, and distributions for sensitivity analysis.

Parameter	Value(range)	Distribution	Reference
Log-logistic OS survival model of Nivolumab plus Gemcitabine-Cisplatin	γ=1.428; θ=23.1912	NA	NA
Log-logistic OS survival model of Gemcitabine-Cisplatin	γ=1.5197; θ=18.479	NA	NA
Generalized gamma PFS survival model of Nivolumab plus Gemcitabine-Cisplatin	γ=1.9887, σ=1.1387, Q=-0.6604	NA	NA
Log-logistic PFS survival model of Gemcitabine-Cisplatin	γ=1.889; θ=7.165	NA	NA
HR for OS (nivolumab plus gemcitabine-cisplatin vs gemcitabine-cisplatin)	0.78(0.63,0.96)	Lognormal	(9)
HR for PFS (nivolumab plus gemcitabine-cisplatin vs gemcitabine-cisplatin)	0.72(0.59,0.88)	Lognormal	(9)
Health state utility			
Utility of PFS	0.80(0.56-1.00)	Beta	(34)
Utility of PD	0.71(0.50-0.92)	Beta	(34)
Disutility of AEs			
Anemia	0.07(0.05-0.09)	Beta	(18)
Neutropenia	0.09(0.06-0.12)	Beta	(18)
Decreased neutrophil count	0.20(0.14-0.26)	Beta	(18)
Decreased platelet count	0.05(0.04-0.07)	Beta	(18)
Decreased white-cell count	0.20(0.14-0.26)	Beta	(18)
Thrombocytopenia	0.05(0.04-0.07)	Beta	(18)
Probability of AEs in Nivolumab group			
Anemia	0.220(0.154-0.286)	Beta	(9)
Neutropenia	0.188(0.132-0.244)	Beta	(9)
Decreased neutrophil count	0.145(0.102-0.189)	Beta	(9)
Decreased platelet count	0.076(0.053-0.099)	Beta	(9)
Decreased white-cell count	0.099(0.069-0.129)	Beta	(9)
Thrombocytopenia	0.066(0.046-0.086)	Beta	(9)
Probability of AEs in Chemotherapy group			
Anemia	0.177(0.124-0.230)	Beta	(9)
Neutropenia	0.153(0.107-0.199)	Beta	(9)
Decreased neutrophil count	0.111(0.078-0.144)	Beta	(9)
Decreased platelet count	0.049(0.034-0.064)	Beta	(9)
Decreased white-cell count	0.038(0.027-0.049)	Beta	(9)
Thrombocytopenia	0.045(0.032-0.059)	Beta	(9)

HR, hazard ratio; OS, overall survival; AEs, adverse events; PFS, progression-free survival; PD, progressive disease; NA, not applicable.

### 2.4 Cost and utility inputs

This study estimated the lifetime treatment costs for patients, including costs related to drugs, laboratory tests, computer tomography (CT) scans, drug administration, supportive care, terminal care, and management of serious adverse events (**Table 2**). Drug price data were sourced from publicly available price databases in the US or China (22, 23). Laboratory test costs included those for immunohistochemical tests, blood tests, urinalysis, liver function blood test panels, and thyroid function tests. CT costs covered head, chest, and abdominal CT scans. This study considered adverse events of grade 3 or higher with an incidence rate of ≥5%, including anemia, neutropenia, decreased neutrophil count, decreased platelet count, decreased white-cell count, and thrombocytopenia (9) (**Table 1**). Costs for laboratory tests, CT scans, disease management, adverse event management, and supportive care were derived from publicly available databases and published literature (13, 18, 24–33). To calculate the costs of cisplatin and gemcitabine, the average body surface areas for patients in the US and China were set at 1.86 m^2^ and 1.72 m^2^, respectively (18, 29). All costs were adjusted to 2023 values using the consumer price index in the US or China and reported in US dollars, with the exchange rate set at the 2023 average rate of 1 USD = 7.05 Chinese Yuan.

**Table 2:** Basic cost and other parameters: baseline values, ranges, and distributions for sensitivity analysis.

Parameter	Value(range) for the US	Reference for the US	Value(range) for China	Reference for China	Distribution
Drugs costs, $					
Nivolumab (100mg)	3042.60(2129.82-3955.38)	(22)	1312.06(918.44-1705.68)	(23)	Gamma
Gemcitabine (1000mg)	31.07(18.26-43.88)	(22)	71.33(1.68-241.53)	(23)	Gamma
Cisplatin (10mg)	2.19(1.53-4.04)	(22)	1.98(0.90-5.65)	(23)	Gamma
Pembrolizumab (100mg)	5641.20(3948.84-7333.56)	(22)	NA	NA	Gamma
Avelumab (800mg)	7172.52(5020.76-9324.28)	(22)	NA	NA	Gamma
Paclitaxel (100mg)	995.00(108.00-1545.30)	(22)	57.03(17.97-321.51)	(23)	Gamma
Laboratory tests costs per time, $					
Immunohistochemical test	81.71(56.50-134.35)	(31)	58.25(51.93-75.37)	(18)	Gamma
Blood test	10.56(8.45-12.67)	(31)	3.14(2.51-3.77)	(18)	Gamma
Urinalysis	3.17(2.54-3.80)	(31)	0.63(0.50-0.76)	(18)	Gamma
Liver function blood test panel	8.17(6.54-9.80)	(31)	5.92(2.81-9.95)	(24)	Gamma
Thyroid function test	39.16(30.00-500.00)	(31)	20.80(8.61-33.02)	(25)	Gamma
Cost of AEs per event, $					
Anemia	7941.00(5558.70-10,323.30)	(18)	138.75(97.13-180.38)	(18)	Gamma
Neutropenia	13,656.00(9559.20-17,752.80)	(18)	115.01(80.51-149.51)	(18)	Gamma
Decreased neutrophil count	13,656.00(9559.20-17,752.80)	(18)	115.01(80.51-149.51)	(18)	Gamma
Decreased platelet count	27768.61(19,438.03-36,099.19)	(33)	1505.92(1054.14-1957.70)	(18)	Gamma
Decreased white-cell count	13,105.00(9173.50-17,036.50)	(18)	113.34(79.34-147.34)	(25)	Gamma
Thrombocytopenia	11,221.93(7855.35-14,588.51)	(26)	3762.67(2633.87-4891.47)	(13)	Gamma
Other costs, $					
Computer tomography per time	690.00(483.00-897.00)	(32)	71.82(32.31-323.28)	(25)	Gamma
Drug administration per time	336.97(246.95-383.39)	(27)	10.38(6.08-17.02)	(28)	Gamma
Supportive care	1447.79(1164.03-1731.55)	(18)	345.60(91.50-952.50)	(18)	Gamma
Terminal care	11,941.96(8956.47-14,927.45)	(29)	2411.66(936.51-6441.42)	(30)	Gamma
Other					
Body surface area, m^2^	1.86(1.30-2.42)	(29)	1.72(1.20-2.24)	(18)	Normal
Discount rate	0.03(0-0.08)	(17)	0.05(0-0.08)	(18)	Uniform
Time horizon, years	30 (10–30)	NA	30 (10–30)	NA	NA
Willingness-to-pay, $/QALY	150,000	(11–13)	38,043	(14–16)	NA

AEs, adverse events; NA, not applicable; QALY, quality-adjusted life-year.

In calculating patient health outcomes, this study considered the quality of life of patients, where the utility value for the PFS state was set at 0.80, the PD state at 0.71, and death at 0. Disutility values for grade 3 or higher adverse events with an incidence rate of ≥5% were also considered, with data extracted from published literature (18, 34) (**Table 1**).

### 2.5 Sensitivity analysis

We conducted sensitivity analyses to examine the robustness of the model results when parameters changed. Univariate sensitivity analyses were performed to explore the impact of individual parameter variations on the base case results and the price of nivolumab at which the nivolumab group becomes cost-effective. Parameters varied within their 95% CIs or ±30% of their baseline values, while the annual discount rate varied between 0-8%, and the time horizon ranged from 10 to 30 years (**Tables 1**, **2**). In addition, two-way sensitivity analyses were conducted on the utilities of PFS and PD states, as well as the HRs for OS and PFS. In the probabilistic sensitivity analysis, we performed 1000 Monte Carlo simulations to investigate the uncertainty of the model results when all parameters varied simultaneously. Parameters varied according to specific distributions, with the ranges and distributions of the parameters detailed in **Tables 1** and **2**.

### 2.6 Subgroup analysis

To explore the economic outcomes of nivolumab plus gemcitabine-cisplatin in different patient subgroups with urothelial carcinoma in the US or China, we analyzed the cost-effectiveness of the subgroups reported in the CheckMate 901 trial by varying the subgroup-specific HRs for OS and PFS. These subgroups included variations in gender, age, Eastern Cooperative Oncology Group (ECOG) performance-status score, tumor cell PD-L1 expression level, presence of liver metastases, and previous systemic cancer therapy. The HRs for OS and PFS in different subgroups are presented in **Supplementary Table 4**.

## 3 Results

### 3.1 Base-case analysis

The base-case analysis found that first-line treatment with nivolumab in combination with gemcitabine-cisplatin (combination therapy) and gemcitabine-cisplatin (chemotherapy) resulted in survival benefits of 4.238 life-years and 2.979 life-years, respectively, in patients with advanced urothelial carcinoma. Notably, with patients in the nivolumab group gaining an additional 1.259 life-years compared to those in the chemotherapy group (**Table 3**). After considering quality of life, nivolumab plus gemcitabine-cisplatin provided an additional 0.931 QALYs and 0.923 QALYs for the US and Chinese populations, respectively, while also increasing the total cost by $108,838 and $48,001, respectively. This resulted in ICERs for nivolumab plus gemcitabine-cisplatin compared to gemcitabine-cisplatin alone of $116,856/QALY in the US and $51,997/QALY in China, which were below the willingness-to-pay threshold in the US and above the threshold in China, respectively.

**Table 3:** Summary of base-case results.

Strategy	Cost ($)	QALYs	Life-years	Incremental cost	Incremental life-years	Incremental QALYs	ICER ($/QALY)
US setting							
Gemcitabine-cisplatin	95,787	2.174	2.979	NA	NA	NA	NA
Nivolumab plus gemcitabine-cisplatin	204,625	3.105	4.238	108,838	1.259	0.931	116,856
China setting							
Gemcitabine-cisplatin	16,632	2.165	2.979	NA	NA	NA	NA
Nivolumab plus gemcitabine-cisplatin	64,633	3.089	4.238	48,001	1.259	0.923	51,997

QALY, quality-adjusted life-year; ICER, incremental cost-effectiveness ratio; NA, not applicable.

### 3.2 Sensitivity analysis

**Figure 2:**
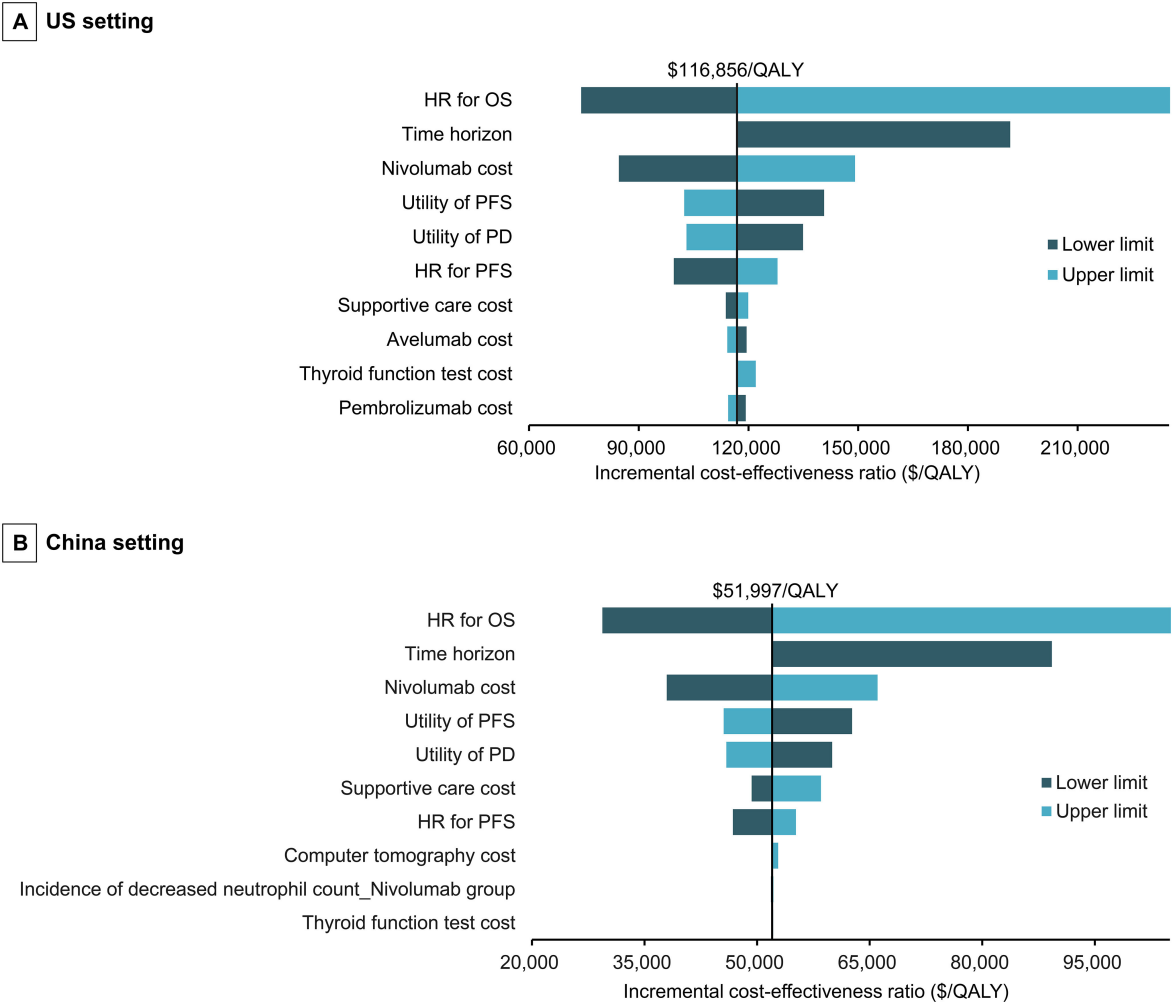
Tornado diagrams of univariable sensitivity analyses. **(A)** US setting; **(B)** China setting. QALY, quality-adjusted life-year; HR, hazard ratio; OS, overall survival; PFS, progression-free survival; PD, progressive disease.

**Figure 3:**
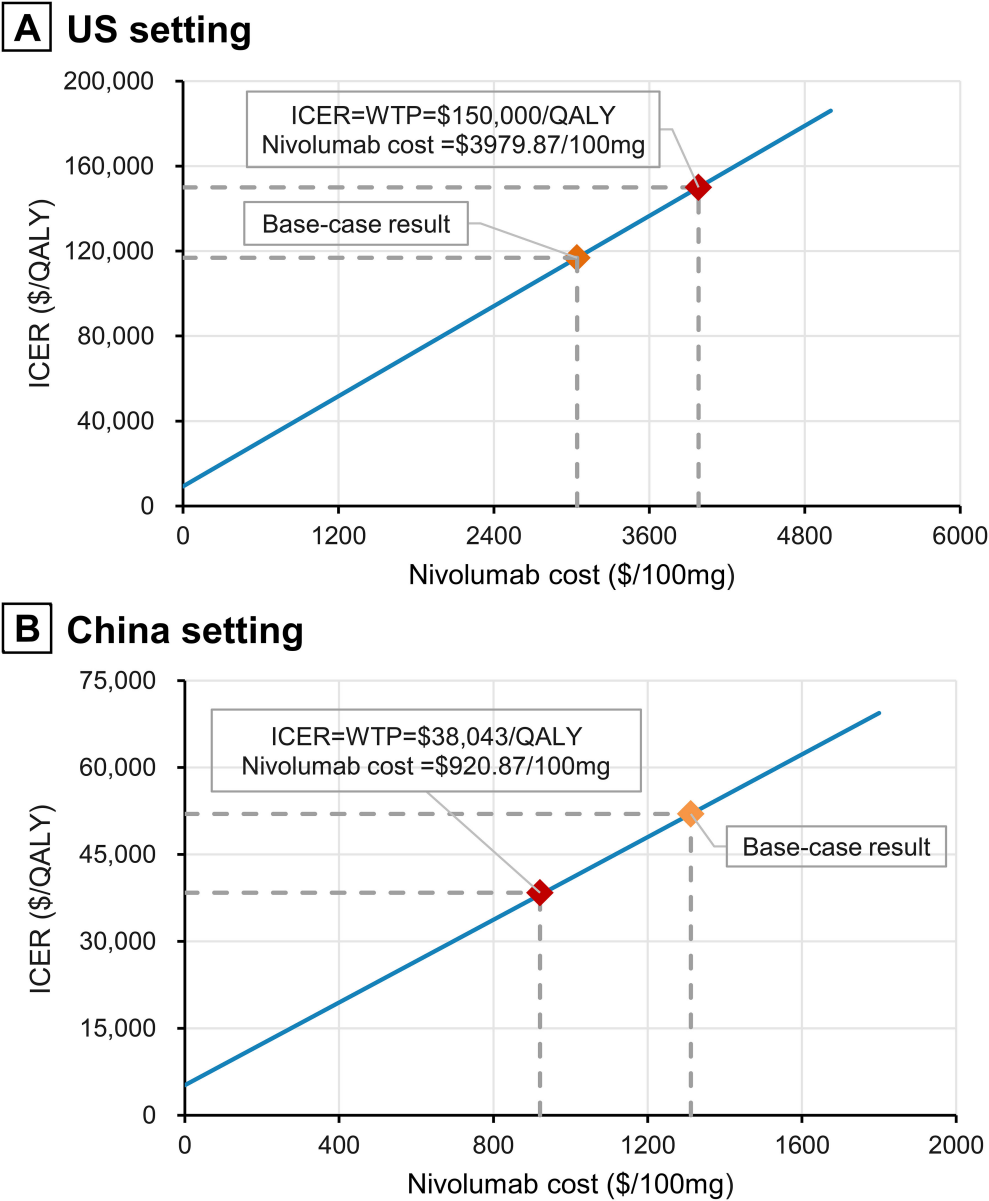
Impact of nivolumab prices on ICERs. **(A)** US setting; **(B)** China setting. QALY, quality-adjusted life-year; ICER, incremental cost-effectiveness ratio.

Univariate sensitivity analysis results revealed that HR for OS, time horizon, and the cost of nivolumab had the greatest impact on ICER (**Figure 2**). In China, the ICER values exceeded the willingness-to-pay threshold when most parameters varied within the set range (**Figure 2B**). In contrast, the ICER remained below the willingness-to-pay threshold for all parameters, except when varying HR for OS and the time horizon in the US (**Figure 2A**). ICER was highly sensitive to the time horizon; at a 5-year horizon, the ICERs for the US and China reached $329,258/QALY and $157,297/QALY, respectively, and gradually decreased with the extension of the time horizon (**Supplementary Figure 2**). Simulation results for the price of nivolumab are shown in **Figure 3** and **Supplementary Table 5**. The ICERs for both the US and Chinese populations decreased with the reduction in the price of nivolumab. When the price of nivolumab in China dropped from the current $1312.06/100mg to $920.87/100mg, nivolumab plus gemcitabine-cisplatin became cost-effective, with price reduction of 29.81% (**Figure 3** **, Supplementary Table 5**). Additionally, the model showed good robustness to changes in other factors such as laboratory test costs and adverse event-related disutilities (**Figure 2**). Univariate sensitivity analysis results for all parameters are presented in **Supplementary Figures 3** and **4**.

Two-way sensitivity analysis results showed that when the utilities of PFS and PD simultaneously increased within the set range, the ICER would decrease but remain above the willingness-to-pay for Chinese populations. When the utilities of PFS and PD fell below 0.63 and 0.57, respectively, the ICER for nivolumab plus gemcitabine-cisplatin exceeded the willingness-to-pay threshold in the US (**Supplementary Figure 5**). However, when the HR for OS and HR for PFS were less than 0.85 and 0.88 respectively, regardless of how the HRs changed, nivolumab plus gemcitabine-cisplatin was cost-effective in the US (**Supplementary Figure 6**). In China, however, the HR for OS and HR for PFS would need to be controlled below 0.74 and 0.88, respectively, for nivolumab plus gemcitabine-cisplatin to achieve cost-effectiveness compared to gemcitabine-cisplatin.

**Figure 4:**
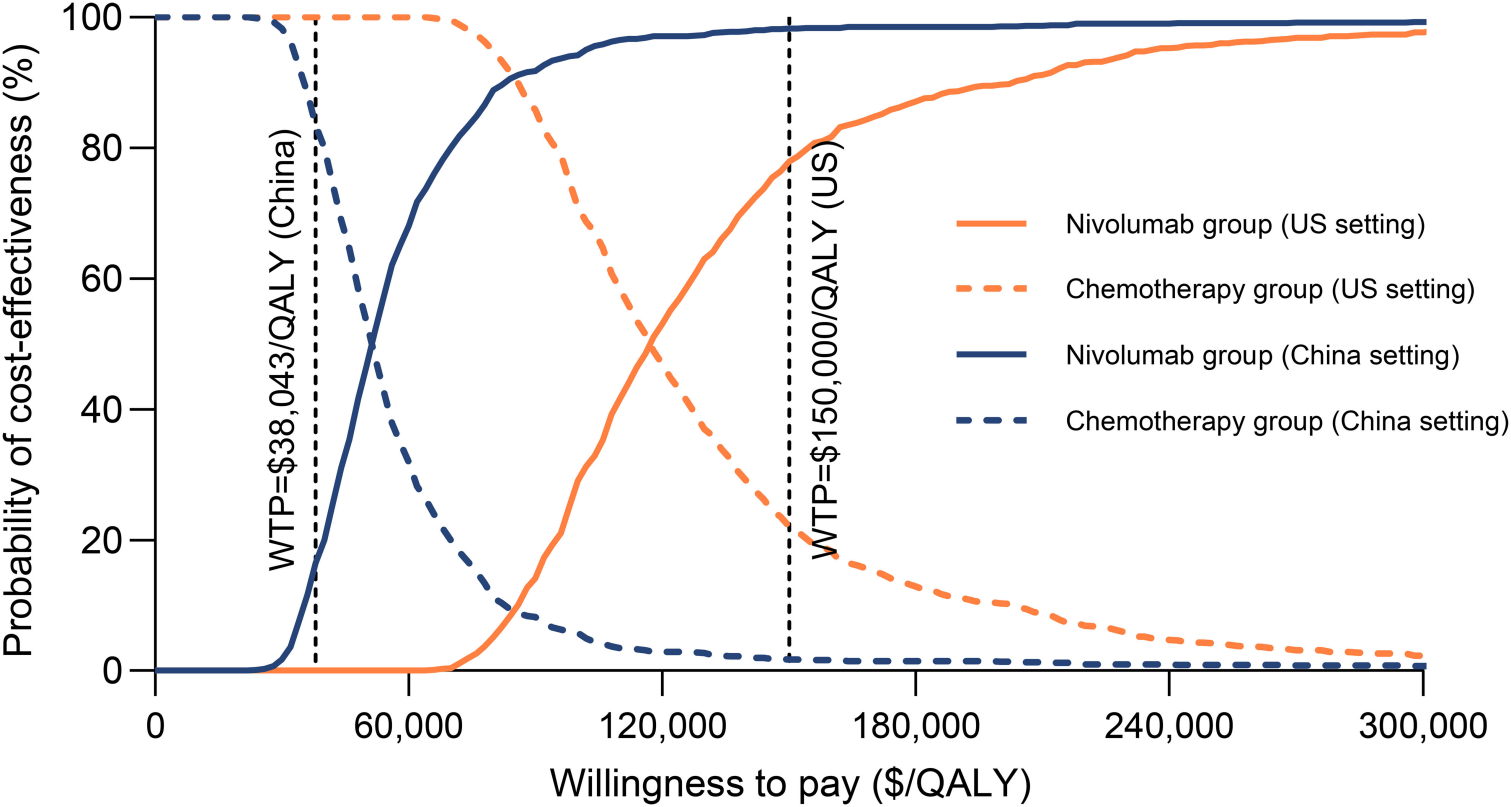
Cost-effectiveness acceptability curves for nivolumab plus gemcitabine-cisplatin vs gemcitabine-cisplatin. QALY, quality-adjusted life-year; WTP, willingness-to-pay.

**Figure 5:**
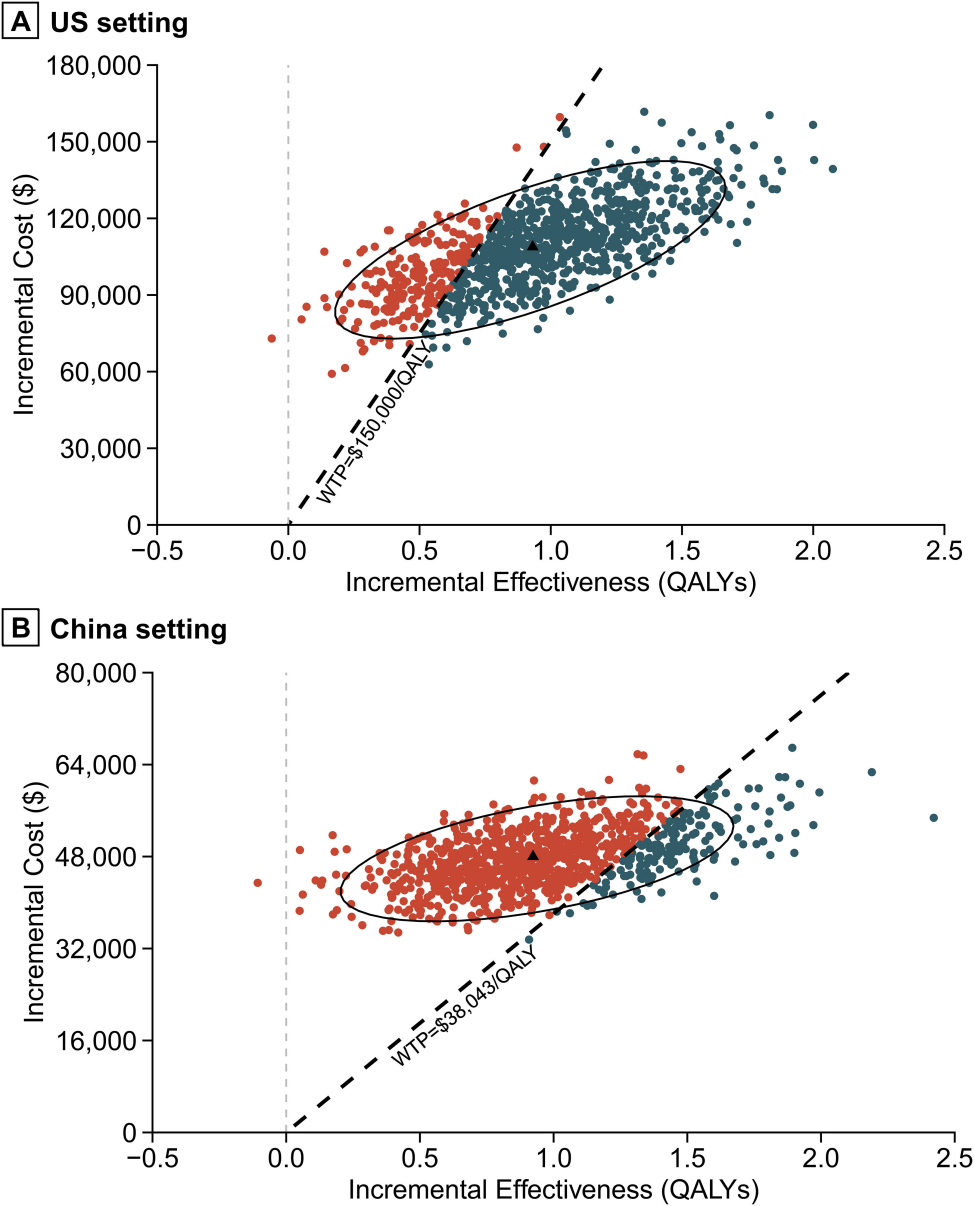
Incremental cost-effectiveness scatter plot. **(A)** US setting. **(B)** China setting. QALY, quality-adjusted life-year.

Probabilistic sensitivity analysis results indicated that, with willingness-to-pay thresholds set at $150,000 per QALY in the US and $38,043 per QALY in China, the probabilities that the combination of nivolumab and gemcitabine-cisplatin being cost-effective compared to gemcitabine-cisplatin alone were 77.5% and 16.5%, respectively (**Figures 4**, **5**). When the willingness-to-pay threshold for China increased to $51,997/QALY, the probability of nivolumab combined with chemotherapy being cost-effective exceeded that of chemotherapy alone.

### 3.3 Subgroup analysis

In the US, the ICERs for nivolumab plus gemcitabine-cisplatin compared to gemcitabine-cisplatin alone fell below the willingness-to-pay threshold for patients under 65 years old, those over 75, and those with an ECOG performance-status score of 0, and those who were previously untreated. The probability of being cost-effective exceeding 50% in these subgroups. This indicated that nivolumab combined with chemotherapy could be considered cost-effective in these patient subgroups. For the Chinese population, the combination of nivolumab and chemotherapy had the highest cost-effectiveness probability in patients with an ECOG performance-status score of 0, under 65 years old, with ICERs for these two subgroups falling below the willingness-to-pay threshold. Additionally, the combination therapy showed a relatively high probability of cost-effectiveness in the subgroup with tumor cell PD-L1 expression ≥1%, but its ICER exceeded the willingness-to-pay threshold (**Table 4**).

**Table 4:** Cost-effectiveness probabilities of nivolumab plus gemcitabine-cisplatin in subgroups.

Subgroup	Sample size		HR for OS (95% CI)	US setting		China setting	
	Nivolumab group	Chemotherapy group		ICER	Cost-effectiveness probability	ICER	Cost-effectiveness probability
Age, years							
<65	150	148	0.69(0.51-0.92)	86,413	96.10%	35,848	59.10%
65 to <75	120	116	0.89(0.63-1.26)	220,023	36.00%	105,752	9.60%
≥75	34	40	0.86(0.49-1.52)	143,598	56.00%	71,983	26.50%
Sex							
Male	236	234	0.76(0.6-0.97)	108,191	82.80%	47,400	24.50%
Female	68	70	0.82(0.54-1.26)	133,655	70.20%	62,179	21.80%
ECOG performance-status score							
0	162	162	0.7(0.51-0.95)	83,217	95.30%	35,605	60.80%
1	140	142	0.85(0.64-1.11)	170,604	41.10%	79,165	5.50%
Tumor cell PD-L1 expression							
≥1%	111	110	0.75(0.53-1.06)	88,746	86.70%	40,788	41.70%
<1%	193	194	0.8(0.62-1.04)	134,879	59.10%	59,785	13.90%
Liver metastases							
Yes	64	64	0.77(0.51-1.16)	125,580	62.80%	53,229	25.50%
No	240	240	0.77(0.61-0.98)	104,810	83.20%	47,370	22.50%
Previous systemic cancer therapy							
Yes	88	68	0.9(0.59-1.38)	206,050	41.60%	103,612	15.60%
No	216	236	0.76(0.6-0.96)	109,818	81.10%	47,868	24.20%

HR, hazard ratio; OS, overall survival; CI, confidence interval; ICER, incremental cost-effectiveness ratio; ECOG, Eastern Cooperative Oncology Group; PD-L1, programmed death-ligand 1.

## 4 Discussion

This study investigated the cost-effectiveness of adding nivolumab to gemcitabine-cisplatin versus gemcitabine-cisplatin alone in advanced urothelial carcinoma, based on results from the CheckMate 901 trial. Our analysis found that combining nivolumab with gemcitabine-cisplatin extended the life expectancy of patients with unresectable or metastatic urothelial carcinoma by 1.259 years, at an additional total cost of $ 108,838 and $ 48,001 for patients in the US and China, respectively. Consequently, ICERs exceeded the willingness-to-pay threshold of $38,043/QALY in China, but below was lower than the cost-effectiveness threshold of $150,000/QALY in the US, suggesting that the addition of nivolumab to chemotherapy was cost-effective for advanced urothelial carcinoma in the US but not in China.

Univariate sensitivity analysis demonstrated that the ICER decreased as the time horizon extended, with the ICER at a 30-year time horizon falling to about one-third of that at the 5-year time horizon, which was the endpoint of the CheckMate 901 trial follow-up. This suggested that nivolumab in combination with gemcitabine-cisplatin could yield more favorable economic outcomes in patients with a longer life expectancy. The results of the price sensitivity analysis indicated that applying price discounts to nivolumab might be the most viable strategy for making the combination treatment cost-effective across all patient populations. Specifically, discount of at least 29.81% in China were required to achieve cost-effectiveness. These findings can inform reimbursement and pricing decisions by public health insurance agencies and private health insurance companies. Moreover, two-way sensitivity analysis revealed that improving the quality of life during treatment for patients with advanced urothelial carcinoma could also enhance the cost-effectiveness of nivolumab combination therapy.

Subgroup analysis validated the results of the sensitivity analysis for HRs related to OS and PFS. Within the context of precision medicine, individualized cancer treatment must consider not only the patient’s physical condition and disease status but also their financial capacity to bear the costs. The subgroup analysis revealed that the combination of nivolumab and chemotherapy exhibited lower ICERs among patients under 65 years of age, males, those with an ECOG performance-status score of 0, tumor cell PD-L1 expression ≥1%, and those who had not received previous systemic therapy for advanced urothelial carcinoma. Furthermore, the combination therapy showed the highest probability of being cost-effective in patients younger than 65 years and those with an ECOG performance-status score of 0. It potentially offers a cost-effective option for patients who are younger than 65 or have an ECOG score of 0 in the US and China. This could inform decision-making for the selection of first-line treatment with nivolumab plus gemcitabine-cisplatin in advanced urothelial carcinoma. Moreover, our study indicated that the cost-effectiveness of nivolumab plus gemcitabine-cisplatin was superior in patients with PD-L1 expression ≥1% compared to those with PD-L1 expression <1%, consistent with previous studies (35, 36). Given the varying association between PD-L1 expression and the efficacy of immune checkpoint inhibitors in urothelial carcinoma patients (37), it is increasingly important to explore the relationship between PD-L1 expression and the cost-effectiveness of these therapies. We recommend increasing patient stratification in clinical trials based on PD-L1 expression levels to provide more detailed information for economic evaluations and clinical individualized drug treatment.

Current cost-effectiveness analyses of first-line immunotherapy treatments for advanced urothelial carcinoma present mixed results. Qin et al. assessed the cost-effectiveness of atezolizumab combined with gemcitabine and platinum-based chemotherapy from the perspective of US payers, based on the IMvigor130 trial, finding it not cost-effective with an ICER of $434,317/QALY (34). Similarly, Hale et al. evaluated the cost-effectiveness of pembrolizumab plus gemcitabine-carboplatin in the US, showing an ICER of $78,925/QALY, which is considered cost-effective under the willingness-to-pay threshold of $100,000/QALY (38). However, Hale et al.’s study was based on the phase 2 clinical trial KEYNOTE-052, and its conclusions require further validation. Furthermore, phase 3 clinical trials KEYNOTE-361 and IMvigor130 failed to demonstrate a significant OS benefit of first-line treatment with pembrolizumab or atezolizumab combined with chemotherapy compared to chemotherapy alone (39, 40). Due to the absence of head-to-head trial data, an analysis comparing the cost-effectiveness of nivolumab against atezolizumab or pembrolizumab has not been conducted. Future research is necessary to explore the cost-effectiveness of different first-line immunotherapies for advanced urothelial carcinoma.

Although this study evaluated the cost-effectiveness of nivolumab plus gemcitabine-cisplatin as first-line treatment for advanced urothelial carcinoma from the U.S. perspective, our findings might still hold relevance for certain European countries, which are also developed nations. Contieri et al. calculated the cost-effectiveness thresholds for five populous European countries (Italy, Spain, Germany, the United Kingdom, and France), which were $106,980, $92,100, $153,600, $144,240, and $130,980, respectively (41). Assuming no differences in the price of cancer drugs or other treatment costs, the probabilities of nivolumab plus gemcitabine-cisplatin being cost-effective in Italy, Spain, Germany, the United Kingdom, and France were 37.5%, 18.8%, 81.3%, 76.1%, and 66.1%, respectively. Moreover, the study by Vokinger et al. showed that the median monthly treatment cost of cancer drugs in the U.S. was 2.31 times higher than in the evaluated European countries (42). When accounting for differences in drug prices but no other cost variations, the ICER for nivolumab plus gemcitabine-cisplatin as first-line treatment for advanced urothelial carcinoma in Europe was $65,474 per QALY, which was below the cost-effectiveness thresholds of the aforementioned European countries. This preliminary analysis suggested that nivolumab plus gemcitabine-cisplatin may be cost-effective as first-line treatment for advanced urothelial carcinoma in some European countries. However, this was an idealized estimate based on price differences with other costs held constant, and actual results would require future analysis using real-world cost data from each country.

This study has several limitations. First, it was based on mathematical modeling using data from the CheckMate 901 phase 3 clinical trial. Given that the longest follow-up period reported in the CheckMate 901 trial was 60.2 months, the extrapolation of OS and PFS data beyond this period employed common methods in economic evaluation, which might diverge from actual outcomes. Second, this study assumed that patients receiving subsequent treatments in the Chinese setting participated in clinical trials as a substitute for pembrolizumab and avelumab therapy. This assumption may have underestimated the drug costs in both groups, potentially leading to biased ICER estimates. Third, the CheckMate 901 trial did not report quality-of-life data; hence, the utility values used in this study were derived from literature and did not account for the disutility of grade 1/2 adverse events, potentially leading to overestimation or underestimation of patient benefits. Additionally, since there are currently no reported utility values for urothelial carcinoma based on the Chinese population, we used utility values from US patients in the China setting. This may result in discrepancies between the ICER for Chinese patients and the actual values. If future clinical studies report health-related quality of life outcomes for the Chinese population, using more reliable utility data could optimize our study’s findings. Lastly, due to the unavailability of raw data, we estimated the costs and effectiveness for each subgroup using subgroup-specific constant HRs for OS and PFS, a common approach in the economic evaluation of oncology drugs. This may introduce bias into the economic results, and caution is advised when interpreting the findings of the subgroup analysis.

## 5 Conclusion

In comparison to gemcitabine-cisplatin, the first-line treatment of unresectable or metastatic urothelial carcinoma with nivolumab plus gemcitabine-cisplatin resulted in ICERs of $116,856/QALY in the US and $51,997/QALY in China. The ICER was below the willingness-to-pay threshold of $150,000 per QALY in the US, indicating that the combination therapy was cost-effective, while in China, the ICER exceeded the threshold of $38,043 per QALY, suggesting that nivolumab plus gemcitabine-cisplatin was not cost-effective. However, this combination therapy may represent a cost-effective option for specific subgroups in China, specifically patients under the age of 65 or those with an ECOG performance-status score of 0. Additionally, a price reduction of at least 29.81% for nivolumab is likely to achieve cost-effectiveness across all patient populations with advanced urothelial carcinoma in China.

## Data Availability

The original contributions presented in the study are included in the article/**Supplementary Material**. Further inquiries can be directed to the corresponding author.
