# Supplementary material for: Cost-effectiveness of nivolumab plus gemcitabine-cisplatin as first-line treatment for advanced urothelial carcinoma in China and the United States
Source: Front Immunol. 2024 Sep 13;15:1426024. doi: 10.3389/fimmu.2024.1426024 (PMC11427378; doi:10.3389/fimmu.2024.1426024)
Supplement: Supplementary file 1 [file DataSheet1.pdf]

Supplementary Material

Supplementary Table 1. CHEERS 2022 checklist

Supplementary Table 2. Summary of subsequent therapeutic drugs and proportion of patients received treatment

Supplementary Table 3. AIC and BIC results for parametric model fitting

Supplementary Figure 1. Original and model-fitted survival curves

Supplementary Table 4. Subgroup-specific OS HRs and PFS HRs

Supplementary Figure 2. Impact of time horizon on ICERs

Supplementary Figure 3. Tornado diagrams of univariate sensitivity analyses for all parameters in the US setting.

Supplementary Figure 4. Tornado diagrams of univariate sensitivity analyses for all parameters in the China setting.

Supplementary Table 5. Nivolumab’s cost-effective price for the first-line treatment of advanced urothelial carcinoma

Supplementary Figure 5. Two-way sensitivity analysis of ICER to utility values in the nivolumab and chemotherapy groups

Supplementary Figure 6. Two-way sensitivity analysis of ICER to HRs for OS and PFS

Supplementary Table 1. CHEERS 2022 checklist

	Item	Guidance for Reporting	Reported in section
**TITLE**			
Title	1	Identify the study as an economic evaluation and specify the interventions being compared.	Title page
**ABSTRACT**			
Abstract	2	Provide a structured summary that highlights context, key methods, results and alternative analyses.	Abstract section
**INTRODUCTION**			
Background and objectives	3	Give the context for the study, the study question and its practical relevance for decision making in policy or practice.	1. Introduction section
**METHODS**			
Health economic analysis plan	4	Indicate whether a health economic analysis plan was developed and where available.	Not Applicable
Study population	5	Describe characteristics of the study population (such as age range, demographics, socioeconomic, or clinical characteristics).	2.2 Patient Cohort section
Setting and location	6	Provide relevant contextual information that may influence findings.	2.1 Model Overview section
Comparators	7	Describe the interventions or strategies being compared and why chosen.	2.2 Patient Cohort section
Perspective	8	State the perspective(s) adopted by the study and why chosen.	2.1 Model Overview section
Time horizon	9	State the time horizon for the study and why appropriate.	2.1 Model Overview section
Discount rate	10	Report the discount rate(s) and reason chosen.	2.1 Model Overview section
Selection of outcomes	11	Describe what outcomes were used as the measure(s) of benefit(s) and harm(s).	2.1 Model Overview section
Measurement of outcomes	12	Describe how outcomes used to capture benefit(s) and harm(s) were measured.	2.1 Model Overview section
Valuation of outcomes	13	Describe the population and methods used to measure and value outcomes.	2.1 Model Overview section
Measurement and valuation of resources and costs	14	Describe how costs were valued.	2.4 Cost and Utility Inputs section
Currency, price date, and conversion	15	Report the dates of the estimated resource quantities and unit costs, plus the currency and year of conversion.	2.4 Cost and Utility Inputs section on pages 8-9
Rationale and description of model	16	If modelling is used, describe in detail and why used. Report if the model is publicly available and where it can be accessed.	2.1 Model Overview section
Analytics and assumptions	17	Describe any methods for analysing or statistically transforming data, any extrapolation methods, and approaches for validating any model used.	2.3 Clinical Efficacy Data Inputs section
Characterizing heterogeneity	18	Describe any methods used for estimating how the results of the study vary for sub-groups.	2.6 Subgroup Analysis section
Characterizing distributional	19	Describe how impacts are distributed across different individuals or adjustments made to	2.5 Sensitivity Analysis section

effects		reflect priority populations.	
Characterizing uncertainty	20	Describe methods to characterize any sources of uncertainty in the analysis.	2.5 Sensitivity Analysis section
Approach to engagement with patients and others affected by the study	21	Describe any approaches to engage patients or service recipients, the general public, communities, or stakeholders (e.g., clinicians or payers) in the design of the study.	Not Applicable
**RESULTS**			
Study parameters	22	Report all analytic inputs (e.g., values, ranges, references) including uncertainty or distributional assumptions.	2.4 Cost and Utility Inputs section
Summary of main results	23	Report the mean values for the main categories of costs and outcomes of interest and summarise them in the most appropriate overall measure.	3.1 Base-Case Analysis section
Effect of uncertainty	24	Describe how uncertainty about analytic judgments, inputs, or projections affect findings. Report the effect of choice of discount rate and time horizon, if applicable.	3.2 Sensitivity Analysis section
Effect of engagement with patients and others affected by the study	25	Report on any difference patient/service recipient, general public, community, or stakeholder involvement made to the approach or findings of the study	4. Discussion section
**DISCUSSION**			
Study findings, limitations, generalizability, and current knowledge	26	Report key findings, limitations, ethical or equity considerations not captured, and how these could impact patients, policy, or practice.	4. Discussion section
**OTHER RELEVANT INFORMATION**			
Source of funding	27	Describe how the study was funded and any role of the funder in the identification, design, conduct, and reporting of the analysis	Funding section
Conflicts of interest	28	Report authors conflicts of interest according to journal or International Committee of Medical Journal Editors requirements.	Conflict of Interest section

Supplementary Table 2. Summary of subsequent therapeutic drugs and proportion of patients received treatment

	Proportion of patients receiving treatment	
**Subsequent therapeutic drugs**	**Nivolumab group**	**Chemotherapy group**
Pembrolizumab	4.6%	17.8%
Avelumab	1.0%	10.5%
Cisplatin	3.6%	5.9%
Gemcitabine	7.6%	5.9%
Paclitaxel	5.3%	8.2%

Supplementary Table 3. AIC and BIC results for parametric model fitting

Parametric models	OS of nivolumab		OS of chemotherapy		PFS of nivolumab		PFS of chemotherapy	
	**AIC**	**BIC**	**AIC**	**BIC**	**AIC**	**BIC**	**AIC**	**BIC**
Exponential	1540.014	1543.731	1614.151	1617.868	1547.910	1551.627	1240.834	1244.551
Weibull	1539.408	1546.843	1610.759	1618.193	1549.748	1557.182	1229.827	1237.261
Gamma	1537.636	1545.070	1609.132	1616.566	1548.750	1556.184	1221.221	1228.655
Gompertz	1541.913	1549.347	1615.479	1622.913	1526.574	1534.008	1242.833	1250.267
Lognormal	1530.243	1537.677	1614.820	1622.254	1496.094	1503.528	1207.076	1214.510
Log-logistic	**1529.130**	**1536.564**	**1605.896**	**1613.330**	1496.263	1503.697	**1200.611**	**1208.045**
Generalized gamma	1531.601	1542.752	1608.836	1619.987	**1486.754**	**1497.905**	1208.315	1219.466

Abbreviations: OS, overall survival; PFS, progression-free survival; AIC, Akaike information criterion; BIC, Bayesian information criterion.

Supplementary Figure 1. Original and model-fitted survival curves. **(A)** Nivolumab group; **(B)** Chemotherapy group.

A

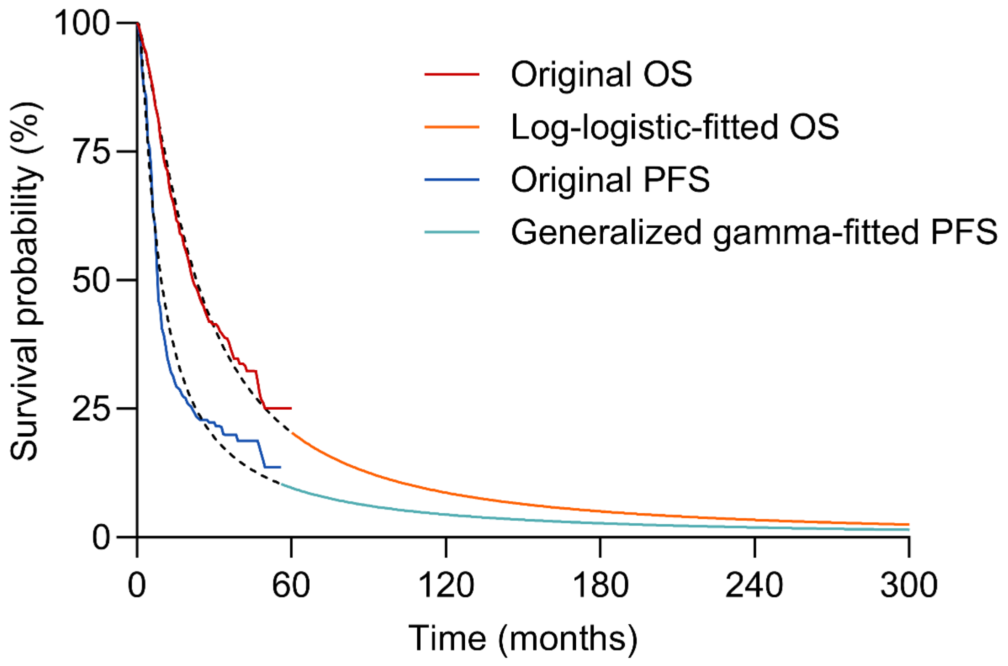

B

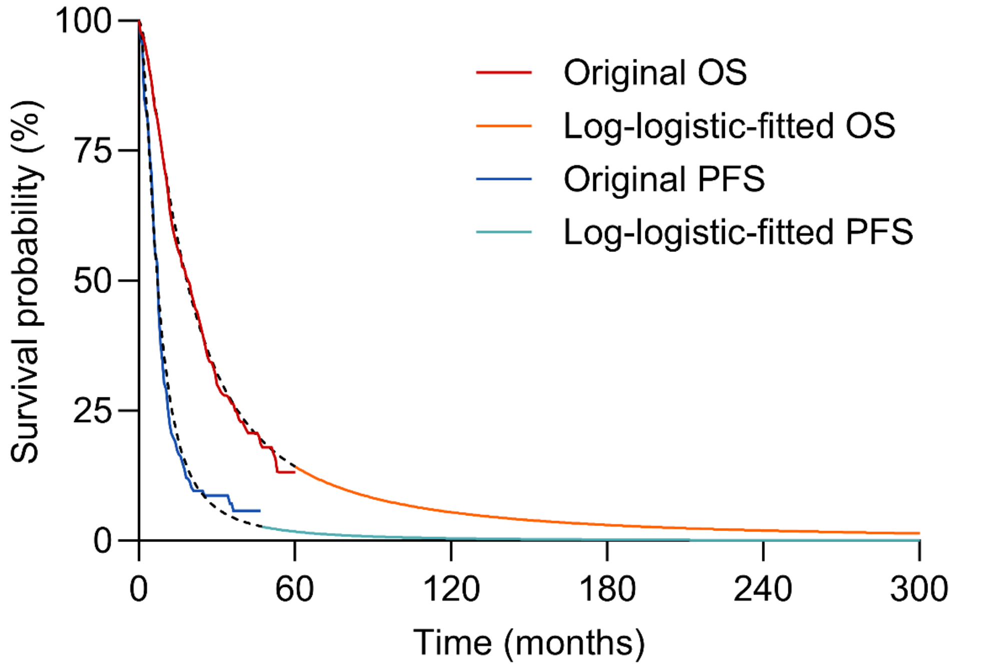

Abbreviations: OS, overall survival; PFS, progression-free survival.

Supplementary Table 4. Subgroup-specific OS HRs and PFS HRs

Subgroup	Sample size			HR for OS (95% CI)			HR for PFS (95% CI)		
	Overall	Nivolumab	Chemotherapy	HR	lower	upper	HR	lower	upper
All patients	608	304	304	0.78	0.63	0.96	0.72	0.59	0.88
Age									
<65 years	298	150	148	0.69	0.51	0.92	0.72	0.54	0.96
65 to <75 years	236	120	116	0.89	0.63	1.26	0.74	0.54	1.02
≥75 years	74	34	40	0.86	0.49	1.52	0.60	0.35	1.01
Sex									
Male	470	236	234	0.76	0.60	0.97	0.72	0.57	0.90
Female	138	68	70	0.82	0.54	1.26	0.68	0.45	1.03
ECOG performance-status score									
0	324	162	162	0.70	0.51	0.95	0.64	0.48	0.86
1	282	140	142	0.85	0.64	1.11	0.76	0.58	1.00
Tumor cell PD-L1 expression									
≥1%	221	111	110	0.75	0.53	1.06	0.58	0.41	0.81
<1% or indeterminate	387	193	194	0.80	0.62	1.04	0.80	0.62	1.02
Liver metastases									
Yes	128	64	64	0.77	0.51	1.16	0.97	0.65	1.45
No	480	240	240	0.77	0.61	0.98	0.65	0.52	0.82
Previous systemic cancer therapy									
Yes	156	88	68	0.90	0.59	1.38	0.65	0.42	1.00
No	452	216	236	0.76	0.60	0.96	0.74	0.59	0.93

Abbreviations: HR, hazard ratio; OS, overall survival; PFS, progression-free survival; ECOG, Eastern Cooperative Oncology Group; PD-L1, programmed death-ligand 1

Supplementary Figure 2. Impact of time horizon on ICERs. **(A)** US setting; **(B)** China setting.

A

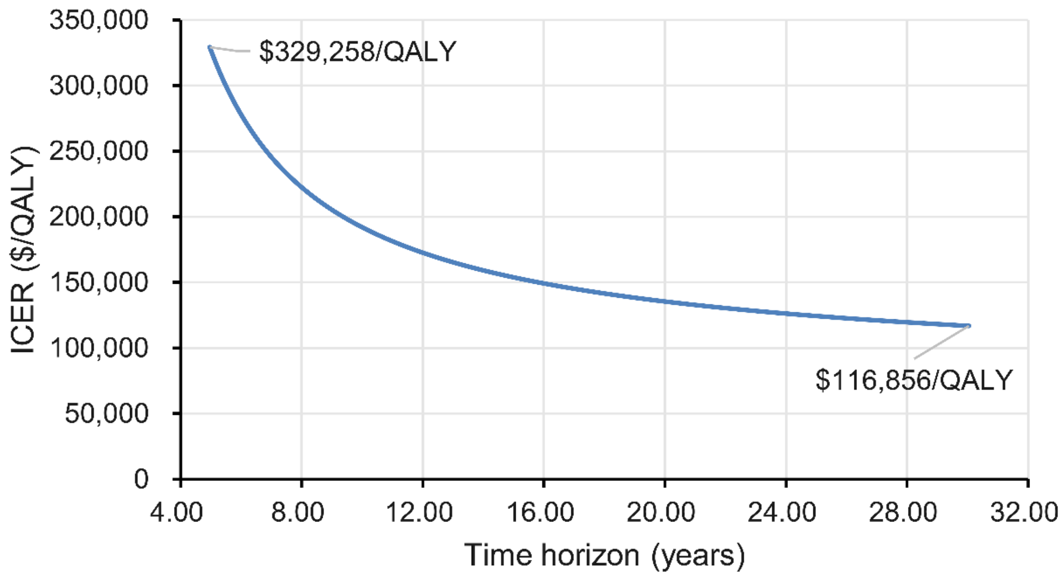

B

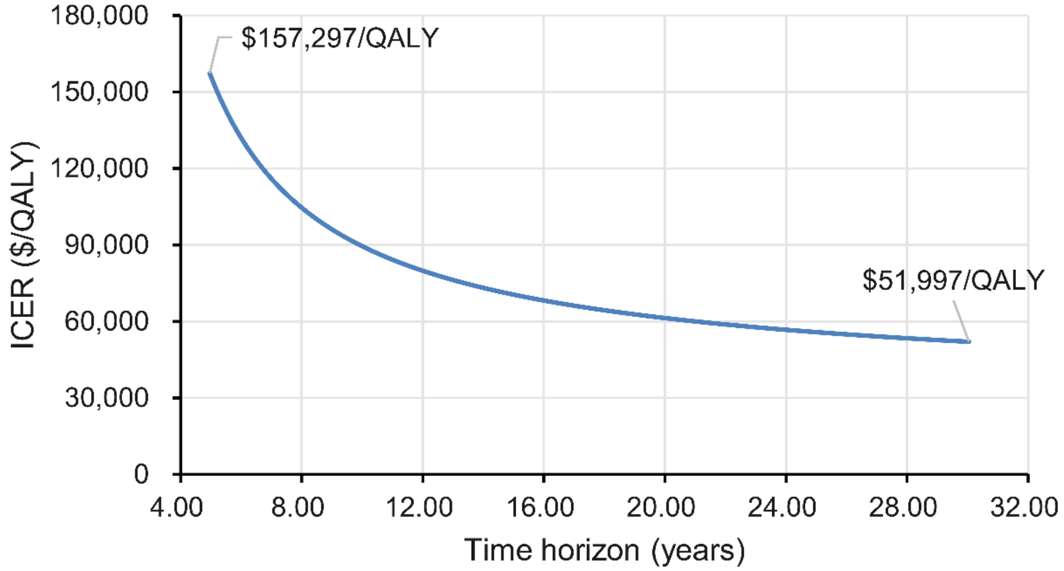

Abbreviations: QALY, quality-adjusted life-year; ICER, incremental cost-effectiveness ratio.

Supplementary Figure 3. Tornado diagrams of univariate sensitivity analyses for all parameters in the US setting.

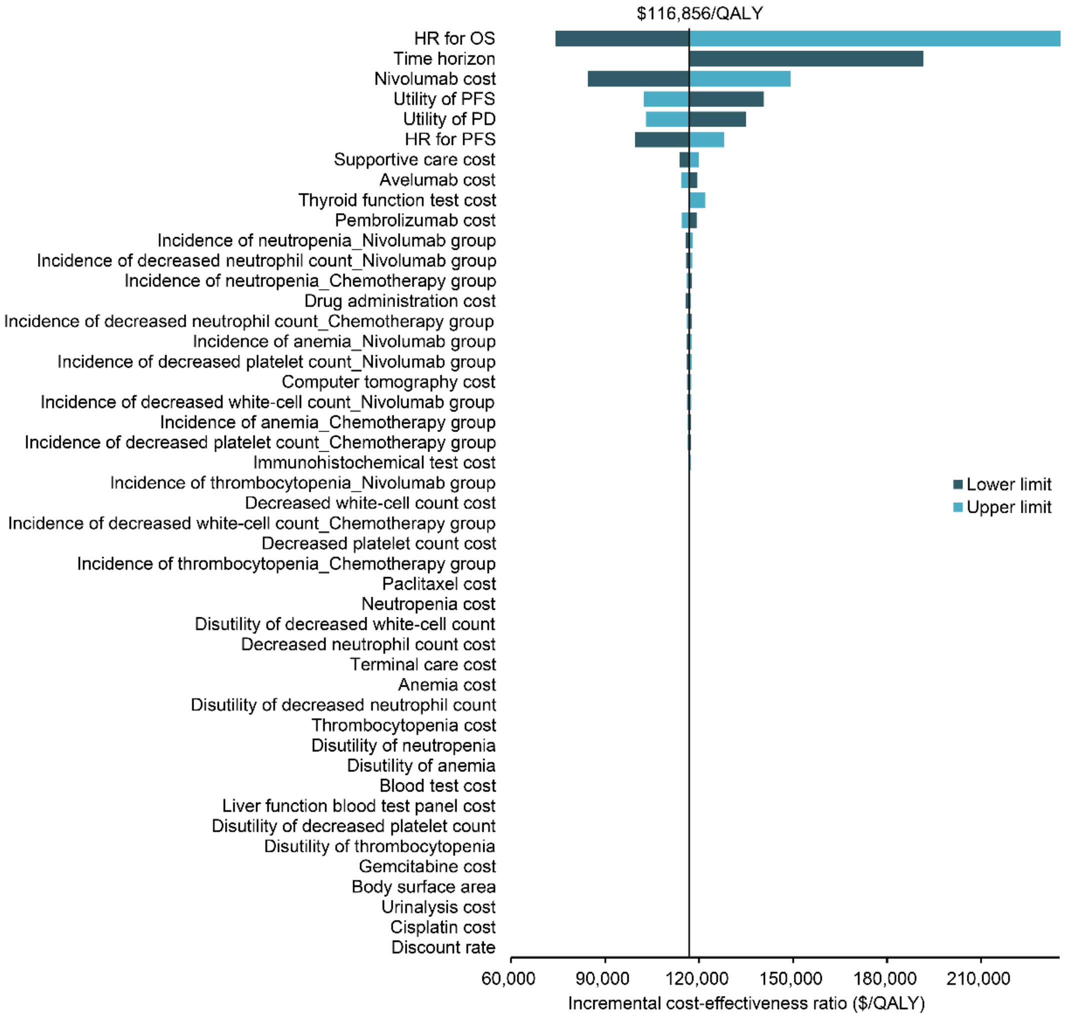

Abbreviations: QALY, quality-adjusted life-year; HR, hazard ratio; OS, overall survival; PFS, progression-free survival; PD, progressive disease.

Supplementary Figure 4. Tornado diagrams of univariate sensitivity analyses for all parameters in the China setting.

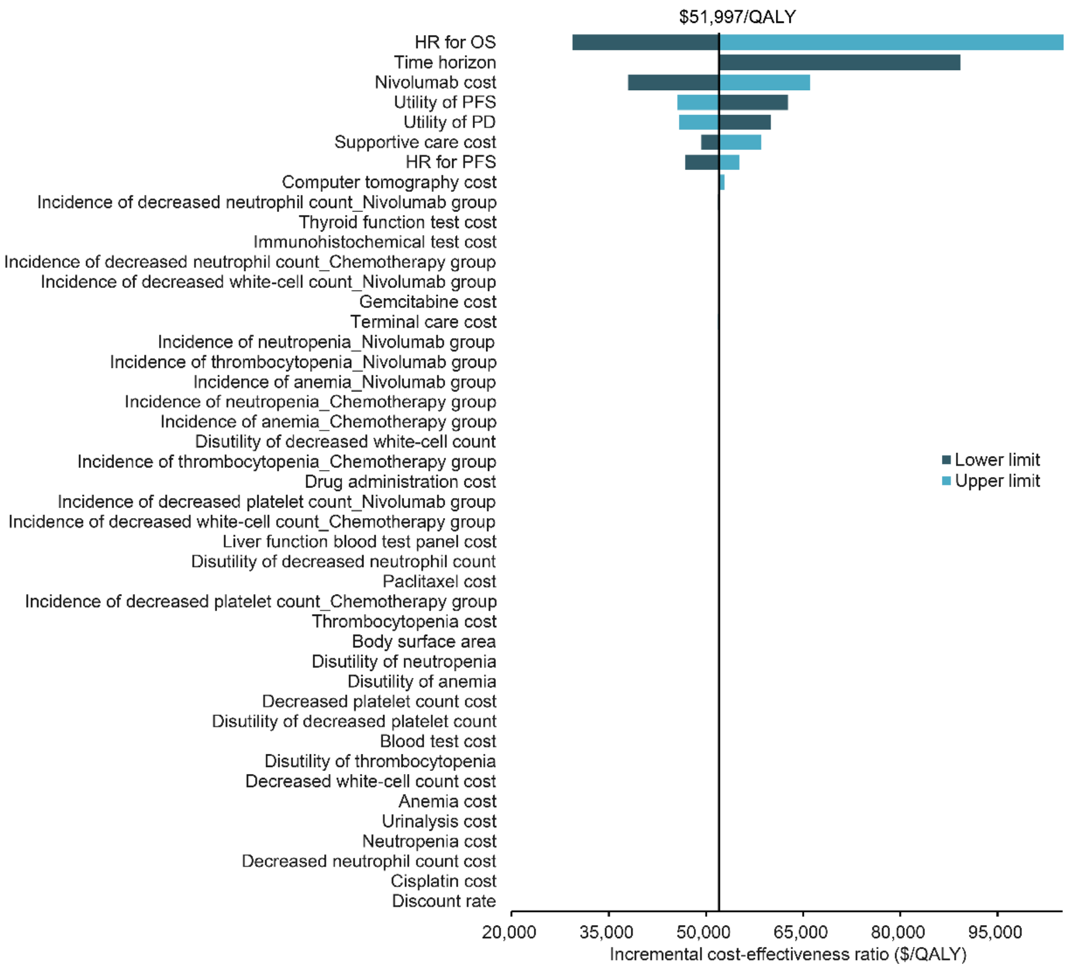

Abbreviations: QALY, quality-adjusted life-year; HR, hazard ratio; OS, overall survival; PFS, progression-free survival; PD, progressive disease.

Supplementary Table 5. Nivolumab's cost-effective price for the first-line treatment of advanced urothelial carcinoma

Country	Willingness-to-pay, $/QALY	Current cost, $/100mg	Cost threshold for cost-effectiveness, $/100mg	Cost required reduction, $/100mg
US	150,000	3042.6	3979.87	-
China	38,043	1312.06	920.87	391.19 (29.81%)

Abbreviation: QALY, quality-adjusted life-year.

Supplementary Figure 5. Two-way sensitivity analysis of ICER to utility values in the nivolumab and chemotherapy groups. **(A)** US setting; **(B)** China setting.

A

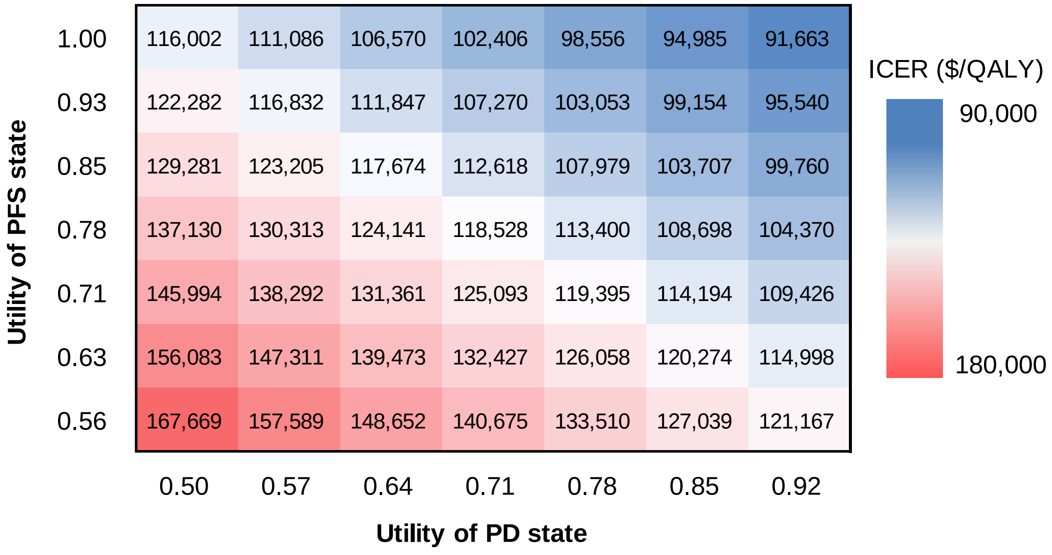

B

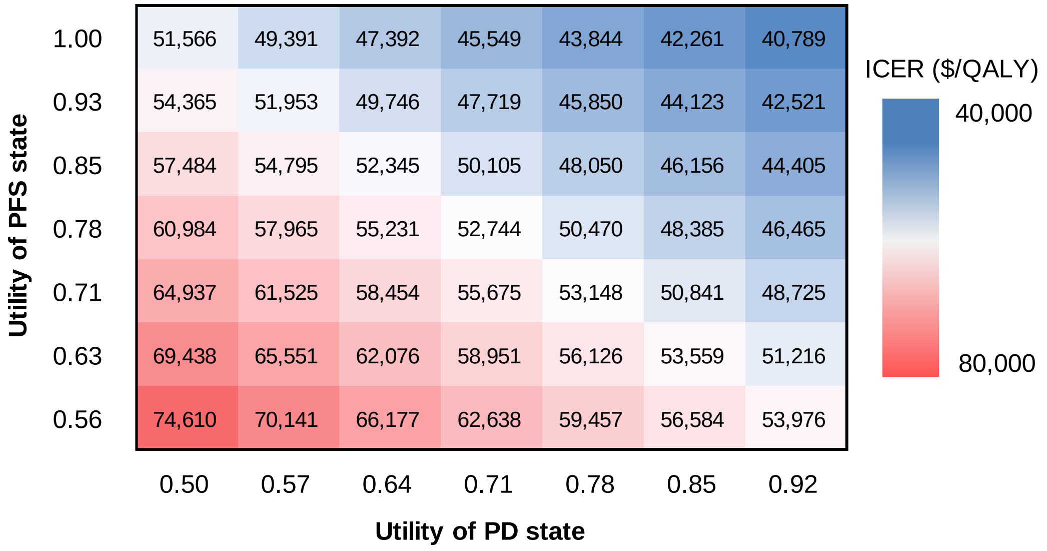

Abbreviations: ICER, incremental cost-effectiveness ratio; PFS, progression-free survival; PD, progressive disease; QALY, quality-adjusted life-year.

Supplementary Figure 6. Two-way sensitivity analysis of ICER to HRs for OS and PFS. **(A)** US setting; **(B)** China setting.

A

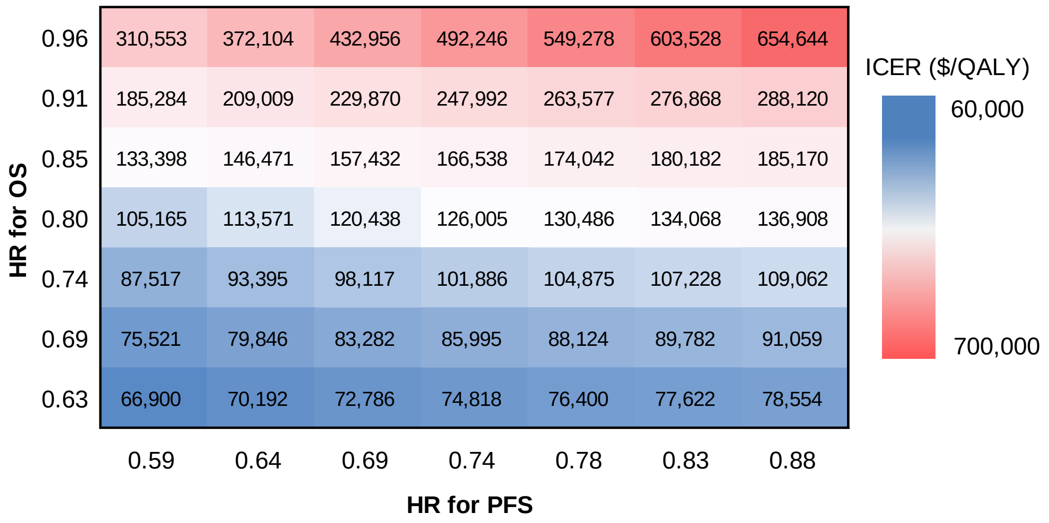

B

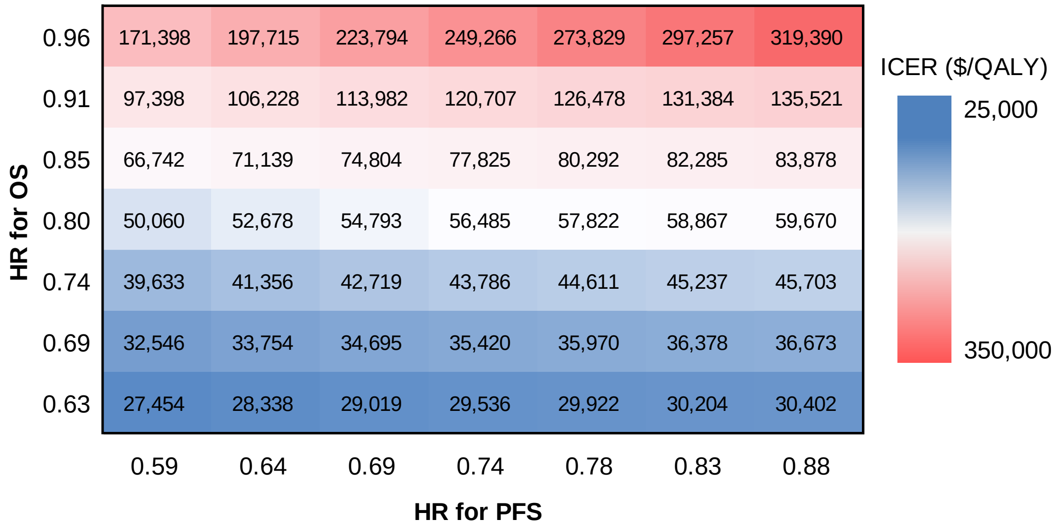

Abbreviations: HR, hazard ratio; OS, overall survival; PFS, progression-free survival; QALY, quality-adjusted life-year; ICER, incremental cost-effectiveness ratio.
